# Vasopressinergic Activity of the Suprachiasmatic Nucleus and mRNA Expression of Clock Genes in the Hypothalamus-Pituitary-Gonadal Axis in Female Aging

**DOI:** 10.3389/fendo.2021.652733

**Published:** 2021-08-24

**Authors:** Angela Cristina Nicola, Larissa Brazoloto Ferreira, Milene Mantovani Mata, Tatiane Vilhena-Franco, Cristiane Mota Leite, Andressa Busetti Martins, José Antunes-Rodrigues, Maristela Oliveira Poletini, Rita Cássia Menegati Dornelles

**Affiliations:** ^1^ Programa de Pós-Graduação Multicêntrico em Ciências Fisiológicas—SBFis/UNESP, Department of Basic Sciences, Araçatuba, Brazil; ^2^University of Sao Paulo (USP), School of Medicine of Ribeirão Preto, Department of Physiology, Ribeirão Preto, Brazil; ^3^University of Northern Paraná (UNOPAR), Londrina, Brazil; ^4^Programa de Pós-Graduação Multicêntrico em Ciências Fisiológicas—SBFis/UEL, Department of Physiological Sciences, Londrina, Brazil; ^5^Federal University of Minas Gerais (UFMG), Institute of Biological Sciences, Department of Physiology and Biophysics, Belo Horizonte, Brazil; ^6^São Paulo State University (UNESP), School of Dentistry, Department of Basic Sciences, Araçatuba, Brazil

**Keywords:** suprachiasmatic nucleus, clock genes, aging, hypothalamus-pituitary-gonadal axis, kisspeptin, vasopressin

## Abstract

The important involvement of the suprachiasmatic nucleus (SCN) and the activity of vasopressinergic neurons in maintaining the rhythmicity of the female reproductive system depends on the mRNA transcription-translation feedback loops. Therefore, circadian clock function, like most physiological processes, is involved in the events that determine reproductive aging. This study describes the change of mRNA expression of clock genes, *Per2*, *Bmal1*, and *Rev-erbα*, in the hypothalamus-pituitary-gonadal axis (HPG) of female rats with regular cycle (RC) and irregular cycle (IC), and the vasopressinergic neurons activity in the SCN and kisspeptin neurons in the arcuate nucleus (ARC) of these animals. Results for gonadotropins and the cFos/AVP-ir neurons in the SCN of IC were higher, but kisspeptin-ir was minor. Change in the temporal synchrony of the clock system in the HPG axis, during the period prior to the cessation of ovulatory cycles, was identified. The analysis of mRNA for *Per2*, *Bmal1*, and *Rev-erbα* in the reproductive axis of adult female rodents shows that the regularity of the estrous cycle is guaranteed by alternation in the amount of expression of *Bmal1* and *Per2*, and *Rev-erbα* and *Bmal*1 between light and dark phases, which ceases to occur and contributes to determining reproductive senescence. These results showed that the desynchronization between the central and peripheral circadian clocks contributes to the irregularity of reproductive events. We suggest that the feedback loops of clock genes on the HPG axis modulate the spontaneous transition from regular to irregular cycle and to acyclicity in female rodents.

## Introduction

The physiological rhythms of development, growth, maturity, and aging are regulated in the suprachiasmatic nucleus (SCN) of the mammalian brain. This nucleus exhibits a marked circadian rhythm in neuronal activity (1, 2) and neurotransmitter synthesis and release (3, 4). In turn, the rhythms are under the regulation of transcription factors generally called clock proteins (5), which are expressed in practically all cell bodies and are the molecular basis of circadian clocks (6, 7). Additionally, the rhythms depend on the activity of vasopressinergic neurons present in the SCN dorsomedial portion, from where they project to important areas in relation to reproduction, such as preoptic area (POA), anteroventral periventricular nucleus (AVPv), dorsomedial hypothalamus (DMH), and arcuate nucleus (ARC) (8). At the cellular levels, inhibitory and stimulatory transcription-translation feedback loops regulate clock proteins. Hormones and signals resulting from the activation of neural networks, such as Kisspeptin neurons, are related to fertility and regularity of reproductive cycles and influence these feedback loops (9, 10). Changes in the pattern of gonadotropin secretion provide powerful information about physiological control mechanisms, such as the underlying characteristics of pulsatile GnRH secretion (11). Furthermore, GnRH neurons reside mainly in POA and ARC, where neurons are sensitive to kisspeptin, steroids, and circadian regulation of AVP (12–14). Over the last decade, a significant body of evidence has accumulated on the interconnections between circadian clocks and aging (15). Among the theories of aging, the neuroendocrine proposes that programmed functional changes in neurons and associated hormones are central to the aging process (16). Studies in physiological aging animal models have demonstrated that there is higher neuronal activity in brain regions known to be involved with reproduction during the transition to reproductive aging (17, 18). The involvement of clock genes in the hypothalamus-pituitary-gonadal axis (HPG) of female rats in the spontaneous transition from regular to irregular cycle and to acyclicity still requires further investigation. It is still unclear to what extent the different physiological phases in life result from effects of the central pacemaker (SCN), peripheral oscillators, both, or on the mechanisms that provide synchronization between the contributing oscillators. In order to characterize the effects of circadian clocks on the aging of the HPG axis, this study evaluated whether the expression of the clock genes on the HPG axis and the vasopressinergic activity of SCN contribute to female reproductive senescence.

## Materials and Methods

### Characterization of the Animal Model

The local Ethics Committee of the Universidade Estadual Paulista approved this protocol for Research Involving Animals (Process n. 2014-00269). The animals were treated according to the laboratory principles of animal care (19). They were housed with *ad libitum* access to food and water. Four-month-old female Wistar rats (adult-regular cycle in diestrus phase), referred to as the regular cycle group (RC), and 18-month IC group (old-irregular cycle in persistent diestrus phase), referred to as the irregular persistent diestrus cycle group (IC), were obtained from the animal facilities of Universidade Estadual Paulista. The analysis of the increase in the duration of the estrous cycle phases, the decrease in phase variability, and the high frequency of days with leukocyte vaginal cytology characterized the irregularity of the estrous cycle in 18-month-old Wistar rats. The animals in the IC group had persistent diestrus lasting 10–12 days longer, with recurrence in three or four cycles. The adult females showed a regular cycle, and the experimental protocols were performed in the diestrus phase. Only adult multiparous rats with regular estrous cycles and senescent rats with irregular estrous cycles and in persistent diestrus participated in this study, presented in at least three consecutive cycles. The experimental procedures during the dark phase were performed using a light emission diode (LED) emission with emission wavelength of 720 nm (red light) and intensity less than 1 lux (20, 21), controlled by a luximeter (Minipa^®^ digital lux meter MLM-1011).

### Collection of Material and Hormone Assays

Rats from RC and IC groups housed under 12/12 h light/dark cycle, lights on at 7:00 h (n=5–10 animals/time/group) were killed by decapitation at 6 h intervals. Blood samples were obtained from the trunk, and brains were rapidly removed and frozen at −70°C.

Blood samples collected in heparinized tubes were centrifuged, and the plasma was stored frozen (−20°C) for hormone assays. Luteinizing hormone (LH) and follicle stimulating hormone (FSH) plasma concentrations were determined using double antibody radioimmunoassay (RIE) with reagents from the National Hormone and Peptide Program (Harbor-UCLA, Torrance, CA, USA). The specific antibodies (anti-rat) used were LH-S10 and FSH-S11 diluted in phosphate buffer with rabbit serum. Standard reference preparations, LH-RP3 and FSH-RP3 ng/ml were diluted in 0.1% phosphate buffer (0.01 M, pH=7.5). After the hormones were iodinated and purified (Celso Rodrigues Franci Laboratory, Medical School of Ribeirão Preto-USP, Brazil), the non-specific antibody was also produced in sheep for precipitation of the reaction in the tests. All samples were dosed in duplicate in the same assay to avoid inter-assay variation. The minimum detectable dose was 0.04 ng/ml for LH and 0.09 ng/ml for FSH. Intra-assay coefficients of variation were 1.8 and 3% for LH and FSH, respectively. The plasma estradiol (E_2_) and progesterone (P_4_) concentrations were measured by Competetive-ELISA using commercial kits (Cayman Chemical Company, MI, USA, for steroids and LSBio, Seattle, USA, for melatonin). E_2_ detection range: 6.6–4.000 pg/ml and sensitivity: 20 pg/ml. P_4_ detection range: 7.8–1.000 pg/ml and sensitivity: 10 pg/ml. All the results were expressed in ng/ml.

### Fixation of Brain Tissue and Double-Immunolabeling Analysis

#### AVP/cFos

RC and IC rats were deeply anesthetized with ketamine (ketamine hydrochloride, Syntec^®^ 80 mg/Kg pc/ip) and xylazine (xylazine-hydrochloride, Syntec^®^ 40 mg/Kg pc/ip) and transcardially perfused with phosphate-buffered saline (PBS), followed by ice-cold 4% paraformaldehyde at 08:00, 14:00, 20:00, or 02:00 on the day of diestrus. Serial coronal brain sections of 30 μm were then cut in four series that represented the anteroposterior length of the SCN. The double-immunolabeling of cFos and argine vasopressin (AVP) was performed on free-floating sections. Sections were first processed for Fos immunoreactivity (anti-Fos antibody, 1:10.000; anti-cFos AB5, PC38, Calbiochem^®^) followed by rabbit anti-AVP (1:20.000, anti-AVP, Bachem T4563). The double labeling were performed using the immunoperoxidase method as described previously (22, 23). Briefly, for cFos, the following were used: anti-rabbit IgG as secondary antibody (1:200; BA 1000, Vector Laboratories), avidin-biotin kit (1:100; Vector Laboratories), cobalt chloride solution 1% (12.5 µl/ml), nickel sulfate solution 1% (1 µl/ml), 0.075 mg/ml 3,3′-diaminobenzidine-HCl (DAB; Sigma-Aldrich, St. Louis, MO, USA), and 0.015% H_2_O_2_ in 0.1 M PB buffer. AVP exposure was performed using DAB (0.075 mg/ml) and H_2_O_2_ (0.3 μl/ml of 30% stock solution in 0.1 M PBS). Next, the sections were mounted on slides (Fisherbrand Superfrost Plus; Fisher Scientific), treated with subbing solution (0.1% gelatin and 0.01% chromium potassium sulfate), and cover slipped with Entellan (Merck^®^).

Immuno-double-stained cells were quantified with the aid of a computerized system that included a Leica microscope (Leica DM 4000B LED) equipped with a Leica digital camera (Leica DFC 450) attached to a contrast enhancement device. Representative sections from three to five animals of each experimental group from similar anatomic levels were analyzed (Bregma −0.60 mm and −0.72 mm were considered) (24). The counting of the double-labeled neurons was performed bilaterally, and neurons with high- and medium-intensity nuclear labeling were considered in this analysis.

#### Kisspeptin/Fos-Related Antigen (FRA)

Sections were incubated with the anti-FRA rabbit antibody (K-25; Santa Cruz Biotechnology, Santa Cruz, CA, USA), at 1:2,000 for 40 h, biotinylated anti-rabbit goat IgG (Vector Laboratories, Burlingame, CA, USA) at 1:600 for 90 min, and avidin-biotin complex solution at 1:100 for 1 h (Elite ABC kit, Vector Laboratories). A solution of nickel sulphate (25 mg/ml), 3,3’-diaminobenzidine-HCl (DAB, 0.2 mg/ml), and 0.03% H_2_O_2_ (Ni-DAB) was used as the chromogen. Then, sections were then incubated with anti-mouse Kp-10 antibody raised in rabbits (A.C. 564), at 1:10,000 for 40 h, biotinylated anti-rabbit goat IgG (Vector Laboratories) at 1:600 for 90 min, and Elite ABC kit at 1:100 for 1 h. DAB solution was used as chromogen. Brain sections were blindly analyzed for experimental groups under a light microscope with an image analysis system (Motic). The number of FRA-, kisspeptin-, and FRA/kisspeptin-immunoreactive (ir) neurons was quantified bilaterally in the ARC (−1.88 to −4.2 from Bregma), according to Paxinos and Watson (24).

### Profile Clock Genes mRNA Expression in HPG Axis

The mRNA quantitative expression for vasopressin (*AVP*), *Period 2* (*Per2*), *Bmal1*, and *Rev-Erbα* was performed in microdissections of the SCN, preoptic area (POA), and medio-basal-hypothalamus (MBH) obtained using the *punch* technique according to Palkovits (25). Coronal brain sections containing the POA, SCN, and MBH according to the Paxinos and Watson (24) atlas were obtained in a cryostat (Leica^®^ 3050S) at −20°C (25). For the POA, a 1,500 µm section was obtained starting at approximately +0.48 mm from the bregma. A single slice of 600 µm, immediately after the POA, was made to the SCN from −0.48 mm posterior to the bregma and two subsequent 1,000 μm slices from −1.72 mm posterior to the bregma for the MBH. The POA and SCN were dissected with 1.5 and 1.0 mm diameter needles, respectively. The SCN and MBH were dissected medially to the third ventricle. The MBH was dissected bilaterally using a 1-mm “square puncher”. All isolated regions were stored in RNA later^®^ (Sigma-Aldrich) solution at −70°C until RNA extraction. After pituitary removal, the adenohypophysis was isolated, stored in RNAse-free Eppendorf tubes, and frozen in liquid nitrogen. Ovaries were isolated from with the adipose tissue and subsequently frozen in liquid nitrogen. The structures were kept at −70°C until RNA extraction.

#### RNA Extraction, cDNA Synthesis, and qPCR

Total RNA was isolated using TRIzol reagent (Invitrogen^®^) according to the manufacturer’s protocol. The RNA concentrations were determined using a Nanodrop 2000c UV-Vis Spectrophotometer (Thermo Scientific). A concentration of 500 ng/µl of RNA was used for cDNA synthesis using the high-capacity complementary DNA reverse transcription kit (Applied Biosystems). Quantitative real-time PCR for *Per2*, *Bmal1*, *Rev*-*erbα*, and *AVP* were performed using a Step One Plus real-time PCR system purchased from Applied Biosystems. The qPCR reactions were performed using two different protocols: TaqMan^®^ for the *Bmal1* gene and SYBR^®^ GreenER™ for *Per2*, *Rev-erbα*, and *AVP*. The access number of each gene, respective primer sequences, and concentrations are shown in **Table 1**. Each experimental cDNA was run in triplicate (2 µl of cDNA per reaction) in 96-well plates. Differences ​​above 0.2 between replicates were automatically excluded. The assays were performed under the following conditions: 10 min at 95°C, followed by 40 cycles of 15 s at 95°C and 1 min at 60°C, 15 s at 95°C, and 1 min at 60°C and 15 s at 95°C, with a gradual increase of 0.3°C. Water (instead of cDNA) was used as a negative control. The housekeeping gene for normalizing *Per2*, *Rev-erbα*, and *AVP* expression was ribosomal 26S RNA, and for *Bmal1* expression was β-actin (**Table 1**). The determination of the gene transcript levels in each sample was obtained using the ΔΔCT method. *Per2*, *Rev-erbα*, and *AVP* relative mRNA level in the unknown sample was calculated using the 2^−ΔΔCt^ method (26). The *Bmal1* relative mRNA level was determined using the relative standard curve method (27) with β-actin as an internal reference. The calibrator group used in both quantifications was CD group/8 h. All results were expressed in arbitrary units of gene expression.

**Table 1:** Sequences and concentrations of primers, and gene access numbers.

Template access numbers	Primers and probes	Final concentration
*m*Per2 (NM_031678.1)	Forward: 5’-GGTCGAGCAAAGGACCGAC-3’ Reverse: 5’-GCTGCTCATGTCCACGTCTT-3’	10 µM
*m*AVP (NM_016992.2)	Forward: 5’-TGCCTGCTACTTCCAGAACTGC-3’ Reverse: 5’-AGGGGAGACACTGTCTCAGCTC-3’	10 µM
*m*Rev-erbα (NM_001113422.1)	Forward: 5’-ACAGCTGACACCACCCAGATC-3’ Reverse: 5’-CATGGGCATAGGTGAAGATTTCT-3’	10 µM
26S RNA	Forward: 5’-CGATTCCTGACAACCTTGCTA-3’ Reverse: 5’-CGTGCTTCCCAAGCTCTATGT-3’	10 µM
*m*Bmal1 (NM_024362.2)	(Rn00577590_m1)	*
*m*ACTB (NM_031144.2)	(Rn00667869_m1)	*

*Inventoried Assay.

### Statistical Analysis

The Shapiro-Wilk normality test was used to verify the normal distribution of data. Statistical differences in qPCR, hormonal levels, and immunohistochemistry assays were determined using two-way ANOVA performed on Graph Pad Prism^®^ software (CA, USA), version 7.0 for Windows, followed by the Tukey post-test for multiple comparisons. The Mann-Whitney test was used to determine differences in body mass. Values are presented as the means ± standard error of mean (S.E.M). P < 0.05 was considered statistically significant for all comparisons.

## Results

### Gonadotropins and Steroids Plasma Concentrations

**Figure 1:**
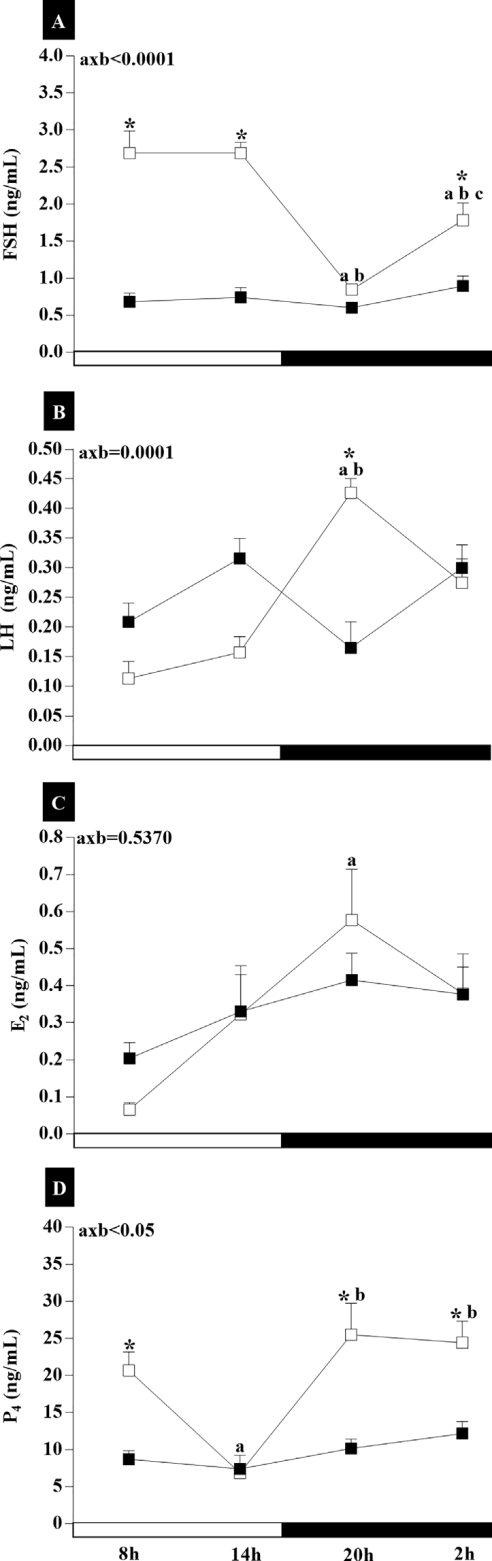
The plasma levels of gonadotropins **(A, B)** and steroids **(C, D)** of adult rats with a regular estrous cycle (RC) on diestrus day (■) and IC group rats with an irregular cycle (IC) in persistent diestrus (□) at 8:00, 14:00, 20:00, and 02:00. The data are expressed as the mean ± SEM. *p < 0.05 *vs.* RC at the same time; ^a^vs. 08:00, ^b^vs.14:00, and ^c^vs. 20:00; axb = interaction of age and time.

**Figure 2:**
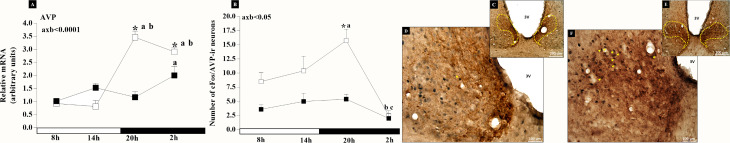
Graphical representation of higher *AVP* gene expression **(A)** and AVP neuronal activity **(B)** observed in rats with irregular cycle (IC group) in persistent diestrus (□) compared to adult rats with regular estrous cycle (RC) on diestrus day (■). The mean ± SEM number of cFos/AVP-ir neurons are shown on the graph. *p < 0.05 *vs.* RC at the same time interval; ^a^vs. 08:00, ^b^vs.14:00, and ^c^vs. 20:00; axb = interaction of age and time. Photomicrographs of vasopressinergic neurons in the dorsomedial portion of the SCN doubly labeled with cFos (arrows) at 20:00 on diestrus day. Animal RC increased by 200× **(C)** and 400× **(D)**; animal IC increased by 200× **(E)** and 400× **(F)**.

To understand the temporal profile of gonadotropin and steroid release in persistent diestrus, analyses of the plasma concentrations of these hormones were made. Statistical analysis of plasma concentrations of FSH and LH indicated an interaction of age and time of day (axb**_FSH_**<0.0001, F_(3,31)_ = 14.13; and axb**_LH_**<0.0001, F_(3,33)_ = 9.245). Temporal analysis revealed no changes in the FSH plasma levels of the RC group (**Figure 1A**). However, the IC group showed higher FSH levels at the light phase compared to dark phase, at 08:00 and 14:00 (p_20h_<0.0001; p_02h_<0.05), as well as those at 02:00 from those at 20:00 (p_02h_<0.05). Intergroup analysis revealed higher plasma FSH concentration in rats in the IC group at 08:00, 14:00, and 02:00 (p<0.0001). The results for LH (**Figure 1B**) in the RC group showed no significant changes in plasma concentrations. For the IC animals, there was a higher plasma concentration of LH at the beginning of the dark phase relative to the light period (p<0.01). RC and IC animals were significantly different at 20:00 (p<0.01). E_2_ plasma concentration (**Figure 1C**) did not show interaction between age and time of day (axb**_E2_** = 0.5370, F_(3,38)_ = 0.7361). IC group showed a considerable increase in the E_2_ plasma concentration until the beginning of the dark phase (p_20h_ <0.05), while in the RC group, basal estradiol levels were observed on diestrus day. In regard to P4 plasma concentrations, there was an interaction between age and time of day (axb_P4_<0.05, F_(3,40)_ = 4.023) (**Figure 1D**). IC rats showed a higher plasma P_4_ concentration, except the 14 h (**Figure 2D**), than RC rats in the diestrus, which showed the lowest levels of P_4_. Statistical differences were observed in the IC group at 14:00 compared to 08:00 (p <0.01), 20:00, and 02:00 (p <0.001). In intergroup comparisons, the differences were found at 08:00 (p <0.05), 20:00, and 02:00 (p <0.001), and there was an interaction between age and time of day (axb**_P4_** <0.05, F_(3,40)_ = 4.023).

These results show that during the spontaneous transition from regular to irregular cycle and to acyclicity, there is greater secretion of FSH and P_4_ and a slight increase in the profile of LH and E_2_ secretion in relation to RC on the day of diestrus.

### Circadian Activity of the AVP Neurons in the SCN Nucleus

This experiment examined the light/dark synchronization of the dorsomedial SCN in persistent diestrus during reproductive aging. Our results showed higher *AVP* gene expression (**Figure 2A**) and AVP neuronal activity (**Figure 2B**) in IC group rats during dark period. RC animals showed higher relative mRNA expression at 02:00 than at 08:00 (*p*<0.05), while in IC group the increase occurred at 20:00 and 02:00, intra- and intergroup (**Figure 2A**). In addition, the IC animals showed greater vasopressinergic activity at 20:00 than at 08:00 (p<0.05) and at 02:00 (p<0.001), as well as at 14:00 relative to at 02:00 (p<0.05) and the intergroup at 20:00 (p<0.01) (**Figure 2B**). The statistical analysis detected that there was an interaction between age and time of day (axb<0.05, F_(3,31)_ = 3.172). In photomicrographs (**Figures 2C–F**), it is possible to check a greater amount of cFos-labeled double vasopressinergic neurons at 20:00 on the day of diestrus in the dorsomedial portion of the SCN of IC rats (**Figures 2E, F**) and a smaller amount in RC rats (**Figures 2C, D**). The results show an increase in SCN dorsomedial neuronal activity, in the dark period, during persistent diestrus.

### Circadian Activity of the Kisspeptin Neuron in the Arcuate Nucleus

**Figure 3:**
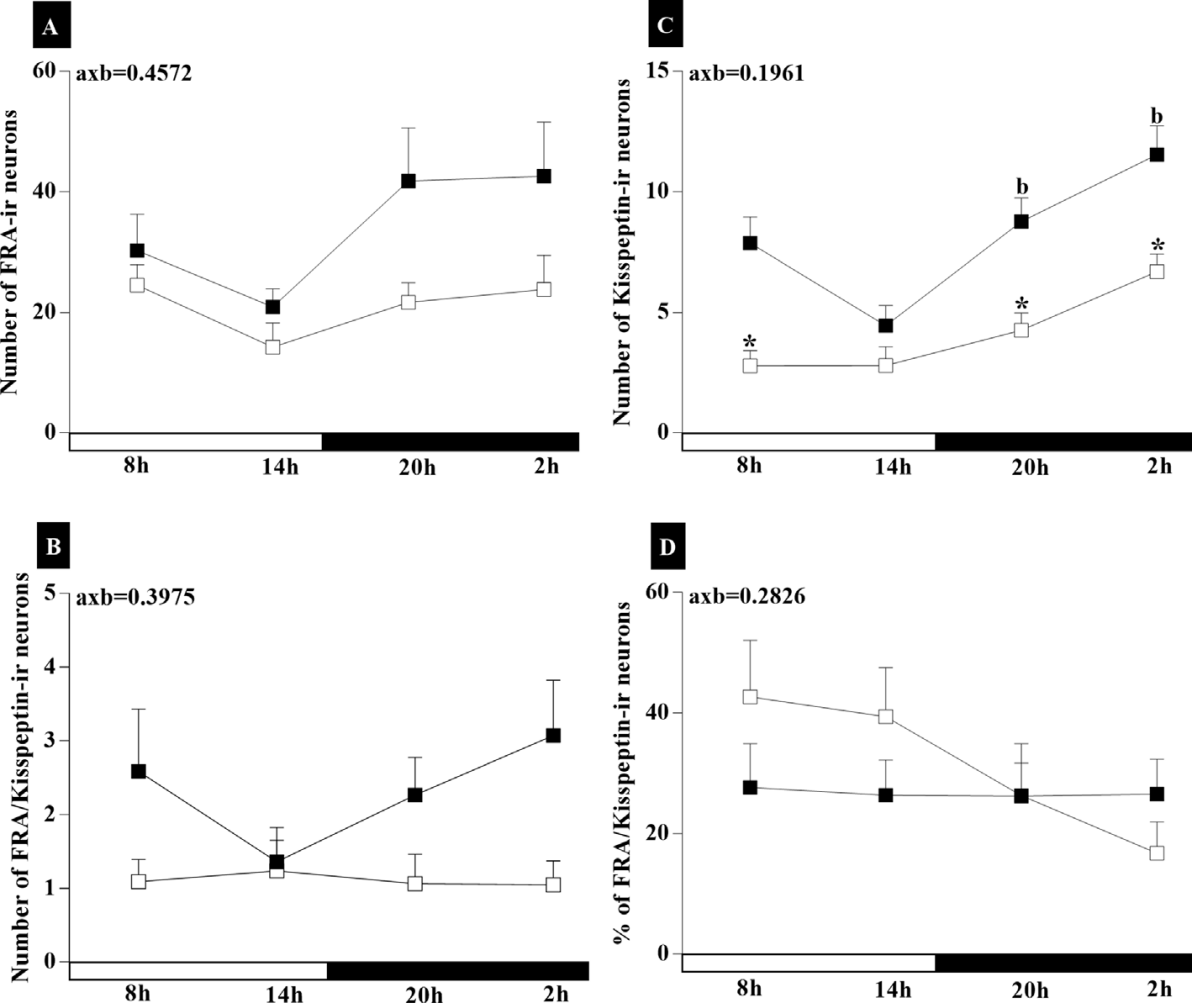
Graphical representation of higher Kisspeptin neuronal activity observed in IC group rats with irregular cycle (IC) in persistent diestrus (□) compared to adult rats with regular estrous cycle (RC) on diestrus day (■). The mean ± SEM number of FRA-ir neurons **(A)**, Kiss-ir neurons **(B)**, FRA/Kiss-ir neurons **(C)**, and % FRA/Kiss-ir neurons **(D)** are shown on the graph. *p < 0.05 *vs.* RC at the same time interval; ^b^vs.14:00; axb = interaction of age and time.

To evaluate whether there is loss of rhythm in the activity of kiss neurons in the ARC nucleus during the circadian cycle of IC animals, analyses of kisspeptin-ir neurons were conducted. Two-way ANOVA showed that there was no interaction between the two variables, groups (RC and IC), and time (8, 14, 20, and 2h) on the number of FRA-ir neurons in the ARC (axb=0.4572, F_3.53_ = 0.8805). There was no statistical difference in the number of FRA-ir neurons in the ARC of both groups (**Figure 3A**). Analysis of kisspeptin-ir neurons (**Figure 3C**) showed that there was no interaction between age (RC and IC) and time of day (axb = 0.1961, F_(3.53)_=1.618). The number of kisspeptin-ir neurons in the ARC was lower (p<0.05) in IC than in RC group at 08:00, 20:00, and 02:00. In the RC group, differences were observed at 20:00 (p<0.05) and 02:00 (<0.0001) in relation to at 14:00. There was no interaction between groups (RC and IC) on the number of FRA/Kiss-ir neurons (axb = 0.3975, F_(3,51)_=1.007), and statistical differences were also not observed in both groups (**Figure 3B**). There was no interaction between groups (RC and IC) and time (8:00, 14:00, 20:00, and 02:00h) on the % of FRA/Kisspeptin-ir neurons in the ARC (axb = 0.2826, F_3.51_ = 1.306) (**Figure 3D**). The results suggest that the circadian signaling of kiss neurons from IC animals is attenuated, contributing to the reduction in the interaction of kiss and GnRH neurons.

### Relative Expression of mRNA Clock Genes in HPG Axis

**Figure 4:**
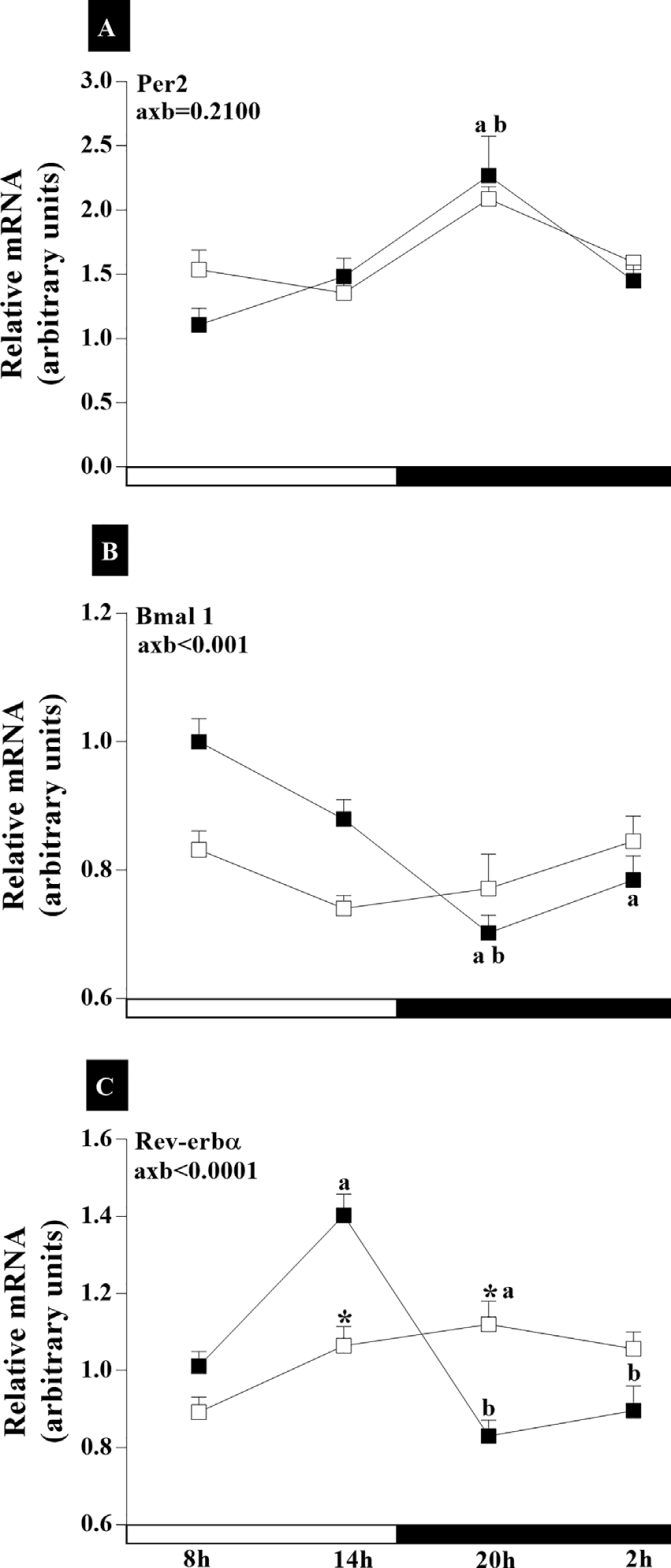
Daily oscillations in relative mRNA expression for *Per2*, *Bmal1*, and *Rev-erbα* in the suprachiasmatic nucleus. Light and dark phases are represented along the X-axis by their respective schedules. Beginning of the light phase at 07:00, when the lights are turned on. The dark phase started at 19:00. All samples were collected at six-hour intervals after the first hour (i.e., 08:00), for 24 h. Light/dark cycle = 12/12 h. **(A)** Relative temporal expression of mRNA for *Per2*, **(B)** *Bmal1*, and **(C)** *Rev-erbα* in animals with a regular cycle on diestrus day and irregular cycle in persistent diestrus. * indicates intergroup variation at the same time: (■) Regular estrous cycle on diestrus day × (□) irregular cycle in persistent diestrus. Letters indicate temporal variation within the group. ^a^vs. 8:00; ^b^vs. 14:00, and ^c^vs. 20:00. axb = age and time of day interaction. n = 3–7. Replicates with a variation above 0.2 were automatically excluded from the groups. ΔΔCt for *Per2* and *Rev-erbα*; relative standard curve for *Bmal1*.

In this analysis, we aimed to verify if there is an alteration in the expression of clock genes in the HPG axis of senescent rats during the spontaneous transition from regular to irregular cycle and to acyclicity, in relation to cyclic female rats. The results demonstrate temporal changes in circadian rhythm amplitude in 18-month-old animals whose cycles are irregular and remain in persistent diestrus. The experiments demonstrated the synchronization of clock gene transcription with gene activity during the SCN light/dark cycle (**Figure 4**). Statistical analyses for *Bmal1*and *Rev-erbα* (**Figures 4B, C**) in the SCN showed an interaction between age and time of day (axb_Bmal1_<0.001, F _(3,105)_ = 5.913; axb_Rev-erbα_<0.0001, F_(3,112)_ = 15.16; and axb_AVP_<0.0001, F_(3, 105)_ = 21.16). There was greater expression of *Per2* at 20:00 (**Figure 4A**) in the SCN of RC groups. The IC group did not present any statistical difference between the dark and light phase schedules. The intergroup comparison also showed no statistical difference (**Figure 4A**). Higher *Bmal1* mRNA were observed in the light phase of RC group compared to the dark phase (p_14x20h_<0.01; p_8x20h_<0.0001; p_8x02h_<0.01) (**Figure 4B**). However, in the IC group, there was no temporal changes in the *Bmal1* mRNA (**Figure 4B**). The expression of *Rev-erbα* mRNA in the SCN of RC group was higher at 14:00 (p<0.0001), decreasing at 20:00 and 02:00 (**Figure 4C**). On the other hand, IC rats showed increases in *Rev-erbα* mRNA expression at 20 h compared at 08:00 (p <0.05) in the SCN (**Figure 4C**). Decreased *Rev-erbα* mRNA occurred at 14:00 h (p<0.01), while it increased at 20:00 h (p<0.01) in the animals of IC group compared to RC group (**Figure 4C**).

**Figure 5:**
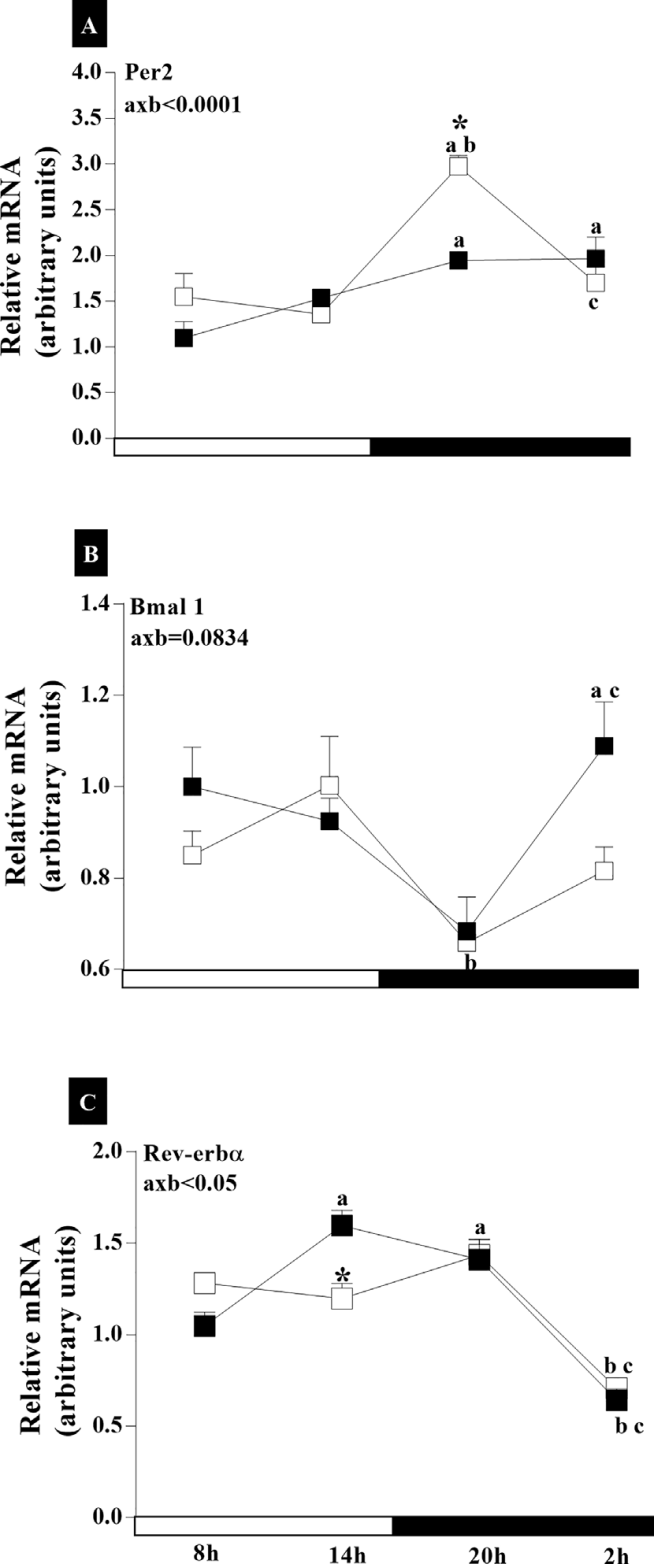
Daily oscillations in relative mRNA expression for *Per2*, *Bmal1*, and *Rev-erbα* in the preoptic area. Light and dark phases are represented along the X-axis by their respective schedules. Beginning of the light phase at 07:00, when the lights are turned on. The dark phase started at 19:00. All samples were collected at six-hour intervals after the first hour (i.e., 08:00), for 24 h. Light/dark cycle = 12/12 h. **(A)** Relative temporal expression of mRNA for *Per2*, **(B)** *Bmal1*, and **(C)** *Rev-erbα* in animals with a regular cycle on diestrus day and irregular cycle in persistent diestrus. * indicates intergroup variation at the same time: (■) Regular estrous cycle on diestrus day × (□) Irregular cycle in persistent diestrus. Letters indicate temporal variation within the group. ^a^vs. 8:00; ^b^vs. 14:00; and ^c^vs. 20:00. axb = age and time of day interaction. n = 3–7; Replicates with a variation above 0.2 were automatically excluded from the groups. ΔΔCt for *Per2* and *Rev-erbα*; relative standard curve for *Bmal1*.

The POA analysis of clock genes mRNA indicated an interaction of age and time of day for *Per2* and *Rev-erbα* (axb_Per2_<0.0001, F_(3,108)_ = 8,098; and axb_Rev-erbα_<0.05, F_(3,130)_ = 5,105) but not for *Bmal1* (axb_Bmal1_ = 0.0834, F_(3,83)_ = 2,299) (**Figure 5A**). In IC group, the greatest *Per2* mRNA was found at 20:00 (p<0.0001). Whereas, in the RC group, *Per2* expression was higher at 20:00 h and 02:00 h compared to 08:00 (p_20h_<0.0001 and p_02h_<0.05) (**Figure 5A**). In the RC group, the *Bmal1* expression was higher at 02:00 h compared to that at 08:00 (p<0.05) and at 20:00 h (p<0.01) (**Figure 5B**). While, in the IC group, lower *Bmal1* expression was found at 20:00 h compared to 14:00 h (p<0.05). In the IC group, *Per2* expression increased at 20:00, while *Rev-erbα* decreased at 14:00 h. *Rev-erbα* in the RC group had higher expression at 14:00 (p_14hx08h_<0.01; p_14hx02h_<0.001) and 20:00 (p_20hx08h_<0.05; p_20hx02h_<0.001) than at 08:00 and 02:00 (**Figure 5C**). On the other hand, the animals with IC expressed a lower amount of *Rev-erbα* mRNA, with similar amounts expressed in the light phase and dark phase, with lower amounts at 02:00 (p_08x02h_<0.0001; p_14x02h_<0.001; p_20x02h_<0.0001), but did not differ from RC rats at this time.

**Figure 6:**
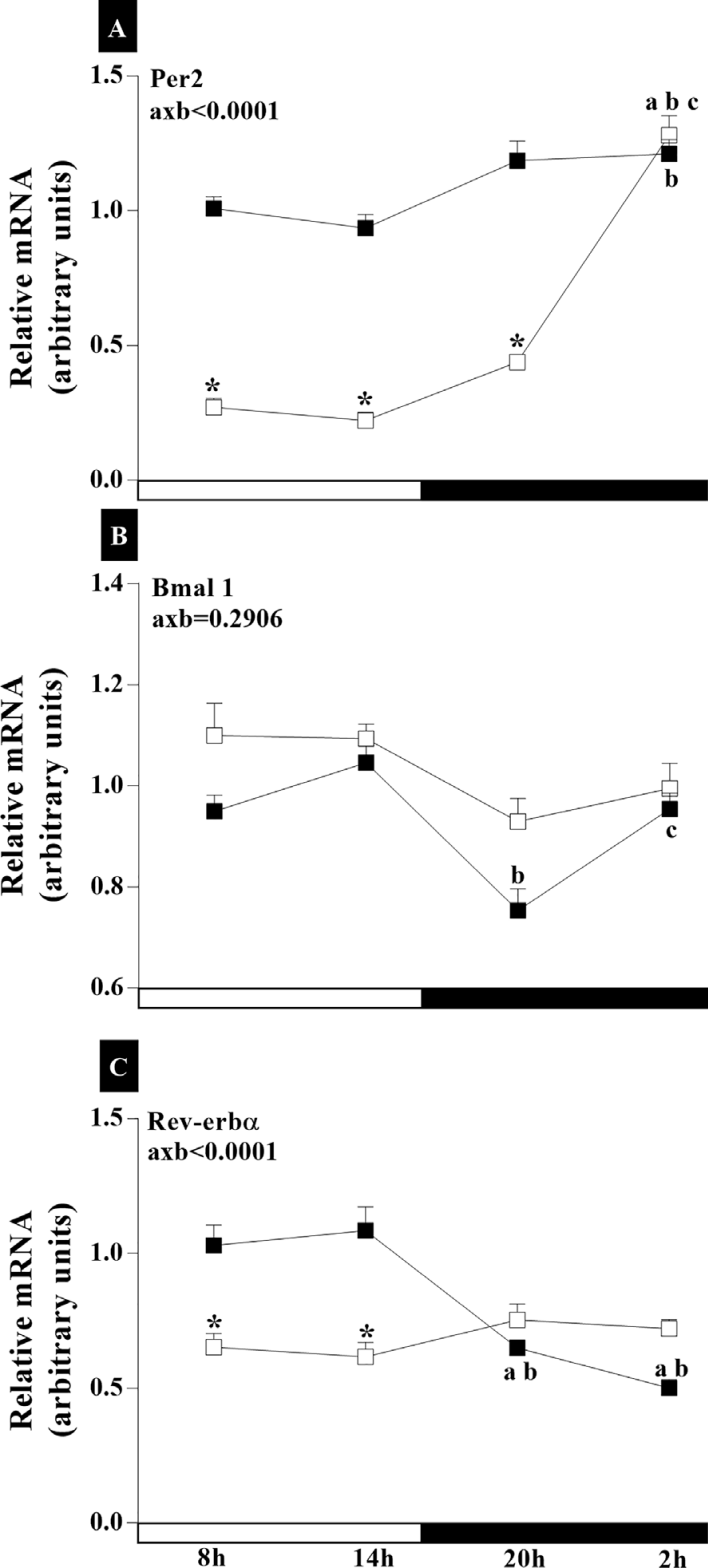
Daily oscillations in relative mRNA expression for *Per2*, *Bmal1*, and *Rev-erbα* in the medio-basal hypothalamus. Light and dark phases are represented along the X-axis by their respective schedules. Beginning of the light phase at 07:00, when the lights are turned on. The dark phase started at 19:00. All samples were collected at six-hour intervals after the first hour (i.e., 08:00), for 24 h. Light/dark cycle = 12/12 h. **(A)** Relative temporal expression of mRNA for *Per2*, **(B)** *Bmal1*, and **(C)** *Rev-erbα* in animals with a regular cycle on diestrus day and irregular cycle in persistent diestrus. * indicates intergroup variation at the same time: (■) Regular estrous cycle on diestrus day × (□) Irregular cycle in persistent diestrus. Letters indicate temporal variation within the group. ^a^vs. 08:00; ^b^vs. 14:00; and ^c^vs. 20:00. axb = age and time of day interaction. n = 3–7. Replicates with a variation above 0.2 were automatically excluded from the groups. ΔΔCt for *Per2* and *Rev-erbα*; relative standard curve for *Bmal1*.

In the MBH, the RC group exhibited slightly higher *Per2* expression at 02:00 h compared to 14:00 h (p<0.01) (**Figure 6A**). In the IC group, there was a decrease in *Per2* mRNA at 8:00 (p<0.0001), 14:00 h (p<0.0001), and 20:00 h (p<0.0001) (**Figure 6A**). *Bmal1* gene expression was higher at 14:00 (p<0.001) and 02:00 (p<0.01) than at 20:00 in the RC group (**Figure 6B**). *Rev-erbα* exhibited higher expression in RC rats at 08:00 and 14:00 than at 20:00 and 02:00 (p<0.05). The IC group showed no time-of-day differences. However, in the group of animals with IC, we found a lower expression of *Rev-erbα* at 08:00 (p <0.01) and 14:00 (p <0.0001) compared to animals with RC (**Figure 6C**). An interaction of age and time of day was observed for analyses of *Per2* (axb<0.0001, F_(3,90)_ = 27) and *Rev-erbα* (axb<0.0001, F_(3,106)_ = 18.04) (**Figure 6**).

**Figure 7:**
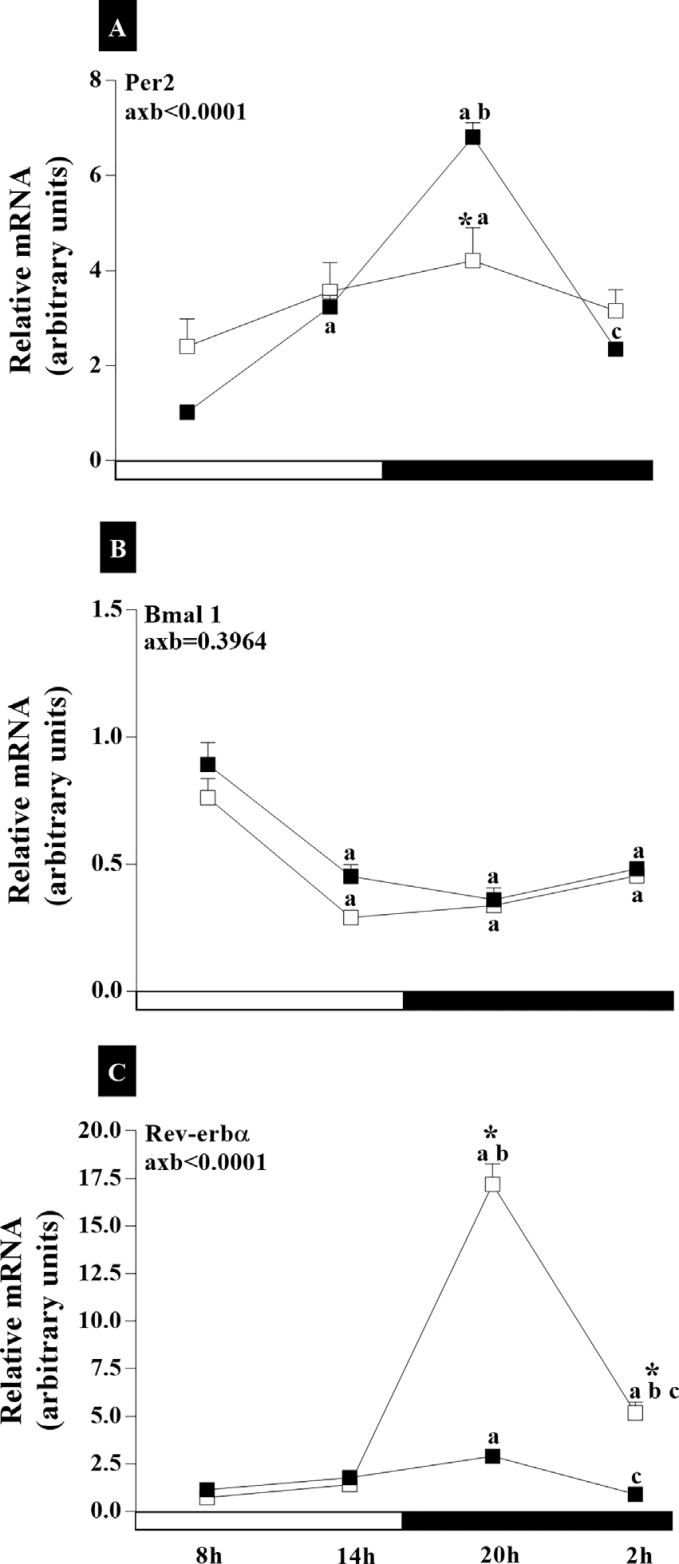
Daily oscillations in relative mRNA expression for *Per2*, *Bmal1*, and *Rev-erbα* in the adenohypophysis. Light and dark phases are represented along the X-axis by their respective schedules. Beginning of the light phase at 07:00, when the lights are turned on. The dark phase started at 19:00. All samples were collected at six-hour intervals after the first hour (i.e., 08:00), for 24 h. Light/dark cycle = 12/12 h. **(A)** Relative temporal expression of mRNA for *Per2*, **(B)** *Bmal1*, and **(C)** *Rev-erbα* in animals with a regular cycle on diestrus day and irregular cycle in persistent diestrus. * indicates intergroup variation at the same time: (■) Regular estrous cycle on diestrus day × (□) Irregular cycle in persistent diestrus. Letters indicate temporal variation within the group. ^a^vs. 08:00; ^b^vs. 14:00; and ^c^vs. 20:00. axb = age and time of day interaction. n = 3–7. Replicates with a variation above 0.2 were automatically excluded from the groups. ΔΔCt for *Per2* and *Rev-erbα*; relative standard curve for *Bmal1*.

In the adenohypophysis, there were significant interactions of age and time-of-day factors on the expression of *Per2* mRNA (axb_Per2_<0.0001, F_(3,76)_ = 10.93) (**Figure 7A**). The RC group showed a peak in *Per2* gene expression at 20:00 h (p_08hx20h_<0.0001; p_14hx20h_< 0.0001), while minimal expression was seen at 08:00 h (**Figure 7A**). In the IC group, *Per2* expression was higher at 20:00 h compared to that at 8:00 h (p<0.05) (**Figure 7A**). However, the expression of *Per2* at 20:00 h (p<0.0001) was lower in senescent females compared to adult female rats (**Figure 7A**). In both RC and IC groups, *Bmal1* mRNA was highest at 8:00 h compared to the times analyzed in this study (**Figure 7B**). For this gene, no interaction of age and time-of-day factors was observed (axb = 0.3964, F_(3,50)_ = 1.009). *Rev-erbα* gene analysis demonstrated a significant interaction of age and time of day (axb<0.001, F_(3,90)_ = 130.3) (**Figure 7C**). The RC group (**Figure 7C**) showed higher *Rev-erbα* expression at 20:00 than at 08:00 and 02:00 (p<0.001). The IC group showed higher *Rev-erbα* expression at 20:00 than at 08:00, 14:00, and 02:00 (p<0.0001). Increase in *Rev-erbα* expression occurred at both times of the dark phase in the IC group (**Figure 7C**).

**Figure 8:**
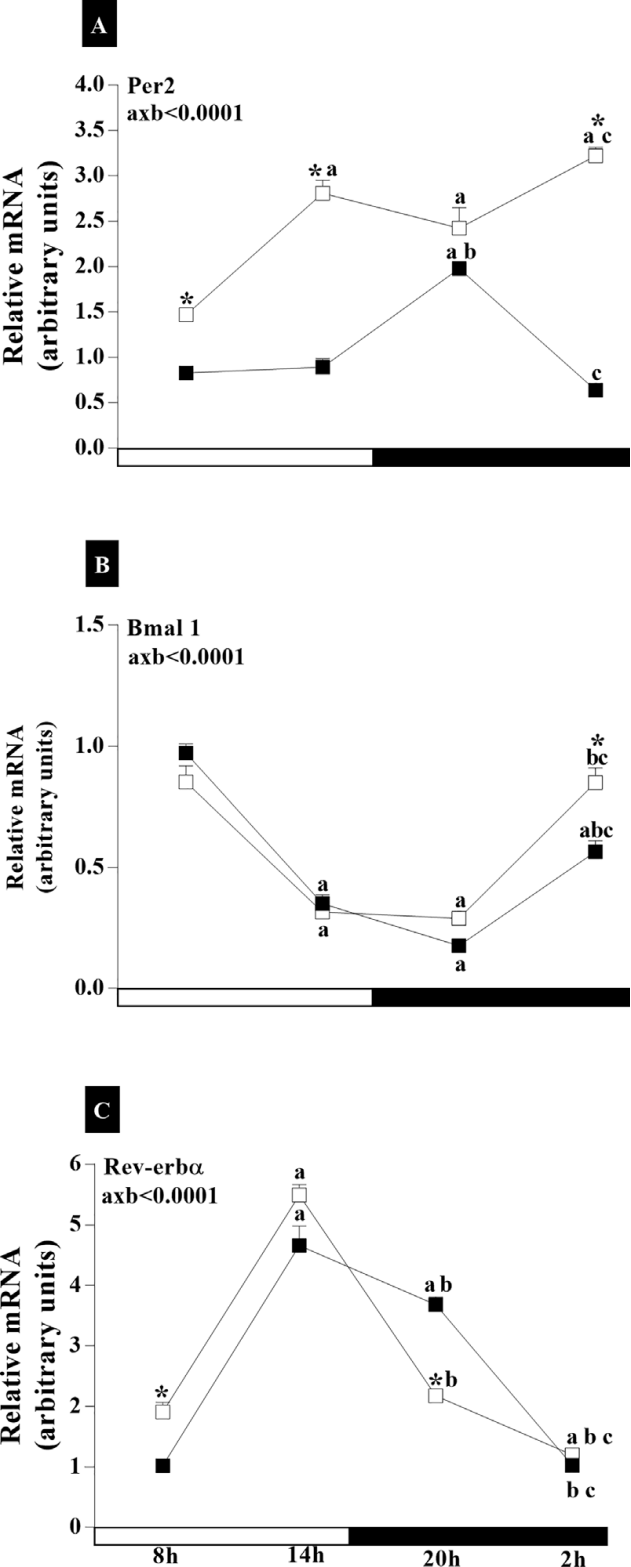
Daily oscillations in relative mRNA expression for *Per2*, *Bmal1*, and *Rev-erbα* in the ovary. Light and dark phases are represented along the X-axis by their respective schedules. Beginning of the light phase at 07:00, when the lights are turned on. The dark phase started at 19:00. All samples were collected at six-hour intervals after the first hour (i.e., 08:00) for 24 h. Light/dark cycle = 12/12 h. **(A)** Relative temporal expression of mRNA for *Per2*, **(B)** *Bmal1*, and **(C)** *Rev-erbα* in animals with a regular cycle on diestrus day and irregular cycle in persistent diestrus. * indicates intergroup variation at the same time: (■) Regular estrous cycle on diestrus day × (□) Irregular cycle in persistent diestrus. Letters indicate temporal variation within the group. ^a^vs. 08:00; ^b^vs. 14:00; ^c^vs. 20:00. axb = age and time of day interaction. n = 3–7. Replicates with a variation above 0.2 were automatically excluded from the groups. ΔΔCt for *Per2* and *Rev-erbα*; relative standard curve for *Bmal1*.

In the ovaries, statistical analyses reveled interaction of age and time of day on *Per2* expression (axb<0.0001, F_(3,71)_ = 38.62), on *Bmal1* expression (axb<0.0001, F _(3,58)_ = 9.448), and on *Rev-erbα* expression (axb<0.0001, F_(3,90)_ = 26.28) (**Figure 8**). The highest level of *Per2* expression in the RC group was at 20:00 (p<0.0001). The IC group showed lower expression at 08:00 h (p<0.0001) and reduced expression at 20:00 h (p<0.0001) (**Figure 8A**). The animals in the IC group showed higher expression of *Per2* at 08:00 h (p <0.05), 14:00 h, and 02:00 h (p <0.0001) than the animals in the RC group (**Figure 8A**). Similar temporal differences in the ovarian *Bmal1* mRNA were found between the RC and IC groups (**Figure 8B**). *Bmal1* expression was greater at 08:00 (p_RC14 and 20h_<0.001 and p_IC14 and 20h_<0.0001) and lower at 14:00 and 20:00 than at 02:00 (p_RC8 and 20h_<0.0001, p_RC14h_<0.05, and p_IC14 and 20h_<0.0001) (**Figure 8B**). There was increased *Bmal1* expression at 02:00 h (p<0.0001) in IC group. There was a significant interaction of age and time of day on *Bmal1* expression (axb<0.0001). The RC and IC groups presented similar gene expression profiles for *Rev-erbα* (**Figure 8C**). There was higher *Rev-erbα* expression ​​at the end of the light phase and the beginning of the dark phase (**Figure 8C**). Increased *Rev-erbα* mRNA at 08:00 h (p<0.01), while it decreased at 20:00 h (p<0.01), occurred in the animals of IC group (**Figure 8C**).

The results showed irregularity in the expression of *Bmal1* and *Per2*, of *Rev-erbα* and *Bmal1* in the HPG axis, between the light and dark phases, in the period of spontaneous transition from regular to irregular cycle and to acyclicity.

## Discussion

In this study, we observed that clock genes expression rates varied between light and dark phases on the HPG axis, as well as SCN neuronal AVP activity in the persistent diestrus of senescent females compared to the diestrus of adult females. Remarkably, these results are important because the synchronization between neural, endocrine, and neuroendocrine signals, coordinated by the SCN and HPG axis, is important for the occurrence and maintenance of reproduction in mammals (28, 29). Also noteworthy is the circadian rhythm for the synthesis activity of vasopressin-releasing neurons in the SCN and its participation in the communication of suprachiasmatic cells. However, the capacity of circadian rhythm regulator genes seems to be diminished during the senescence period. In this sense, studies in the late 1990s showed the importance of the SCN in regulating the circadian system in aged organisms (30–32). We detected higher nocturnal gene expression of *AVP* and neuronal activity in the SCN, mainly in females with irregular estrous cycle and reproductive senescence, when compared to adult female rats in the diestrus phase of the regular cycle. However, there was found a smaller number of kisspeptinergic neurons in the ARC of IC rats. We highlight here the studies carried out by Padilla et al. (33), showing that only female rodents have kisspeptin neurons in the SCN, some of which corresponded to AVP neurons. These authors also showed that the silenced kisspeptin ARC neurons in female mice resulted in the loss of the normal estrous cycle, remaining in diestrus (33, 34). Thus, circadian information from the vasopressinergic and kisspeptinergic neural pathways are essential for the activity of GnRH cells (35–37). Study carried out by our group (38) showed a smaller and constant GnRH content in 18-month-old IC group, similar to the immunostaining data of Kiss neurons in the ARC nucleus. Therefore, our data suggest that communication with GnRH cells *via* kisspeptin in ARC may be compromised and lead to a period of persistent diestrus in IC rats. Furthermore, the SCN analysis showed different expression of *Per2*, *Bmal1*, and *Rev-erbα* in the diestrus of RC rats compared to IC rats with persistent diestrus, especially at 20 h, reinforcing the idea of ​​a weakening of the SCN output system (39). These results together reflect the loss of circadian oscillation, which in RC animals is marked by a reduction in the activity of kisspeptin neurons at the end of the light phase with a consequent increase in the dark phase.

An interesting finding of this study was the higher expression of *Per2* at 20:00 in the POA of animals in the IC group, similar to that observed in the SCN and coinciding with the greater expression and activity of AVP neurons in the SCN. In addition, aged animals show phase misalignment and lack of rhythmic profile in plasma LH secretion, whose concentration was slightly higher in the dark phase, similar to estradiol concentrations. A subpopulation of the GnRH neurons in the MBH expresses endogenous clock functions, and gene expression is necessary for continuous and typical GnRH secretion (40–42). We verified that the relative expression of *Per2* did not change in the MHB of the RC rats in diestrus. However, in IC rats in persistent diestrus, the expression of *Per2* was lower at 8, 14, and 20 h. These data allow us to suggest linearity of gene expression in the MBH related to the linearity of the GnRH content observed in rodent females from the IC group, as verified in a previous study of our group (38). We also found differences in the expression of *Bmal1* and *Rev-erbα* in SCN and MBH in senescent rats. The absence of circadian rhythmicity of these genes leads us to speculate about the physiological activity of the molecular clock as a determining factor for the absence of ovulatory peaks during female aging. In addition to our data, studies have shown that rodent knockouts of the central *Bmal1* gene are infertile and the mutation/deletion of the gene in the periphery contributes to infertility by compromising the production of steroid hormones, gametogenesis, and abnormalities of delivery and implantation in female (43, 44).

Although pituitary cells are under the control of hypothalamic factors, the adenohypophysis cells have a molecular clock capable of measuring time autonomously (45). The molecular clock control in the different cell types of the adenohypophysis provides circadian oscillation in pituitary gonadotrophs, and the daytime rhythm of cell proliferation is synchronized with the estrous cycle in adult rats (29, 46, 47). Our study demonstrates that these cells are the first extra-SCN elements of the HPG axis to signal changes and contribute to reproductive aging. In IC rats, there was no alteration in the expression of *Per2* and *Bmal1*, although we verified a strong inhibition of *Rev-erbα* at the same time (20 h) as there is an increase in LH, E_2_ and P_4_, and lowest in FSH. Interestingly, IC rats exhibit the same response profile for *AVP* mRNA in the SCN, *Rev-erbα* in the adenohypophysis, and plasma LH concentration, suggesting that the non-oscillation in the expression of *Bmal1* and *Rev-erbα* in the SCN at the times analyzed contribute to the increase in the expression of AVP in SCN and plasma LH. In addition, molecular clock components have been detected in the pituitary gland of the rat with autonomous activity and rhythmic LH release, independent of hypothalamic control (48). Perhaps higher plasma E_2_ and P_4_ concentrations in IC animals modulate the axis, as estrogen itself alters circadian rhythms, increasing their amplitude (49, 50).

Reproductive senescence in laboratory rodents is characterized by follicular atresia, irregular cycles, and steroid hormone fluctuations (51). Remarkably, several studies have shown that there are temporal variations in molecular clock components in ovarian rodent tissues, whose function is related to the timing of gene expression in mature granulosa cells, including genes related to steroidogenesis, gonadotropin responsiveness, and ovulation (52–55). Results of the analyses carried out in the diestrus period of RC rats showed the synchrony of the expression of *Per2* and *Bmal1* in the ovary and SCN, reinforcing the important synchronization in the expression of clock genes and the integration in the HPG axis modulating the reproductive system. However, this study shows the desynchronization of the expression of these genes in the ovary and that this desynchronization occurs in a subtle way in relation to the SCN and POA. In these animals, transcription of ovarian genes seems to occur autonomously, with the pattern of response of the *Per2* transcript contrary to that of RC animals, suggesting an ovarian temporal window in response to the central stimulus, contributing to persistence in the diestrus phase.

**Figure 9:**
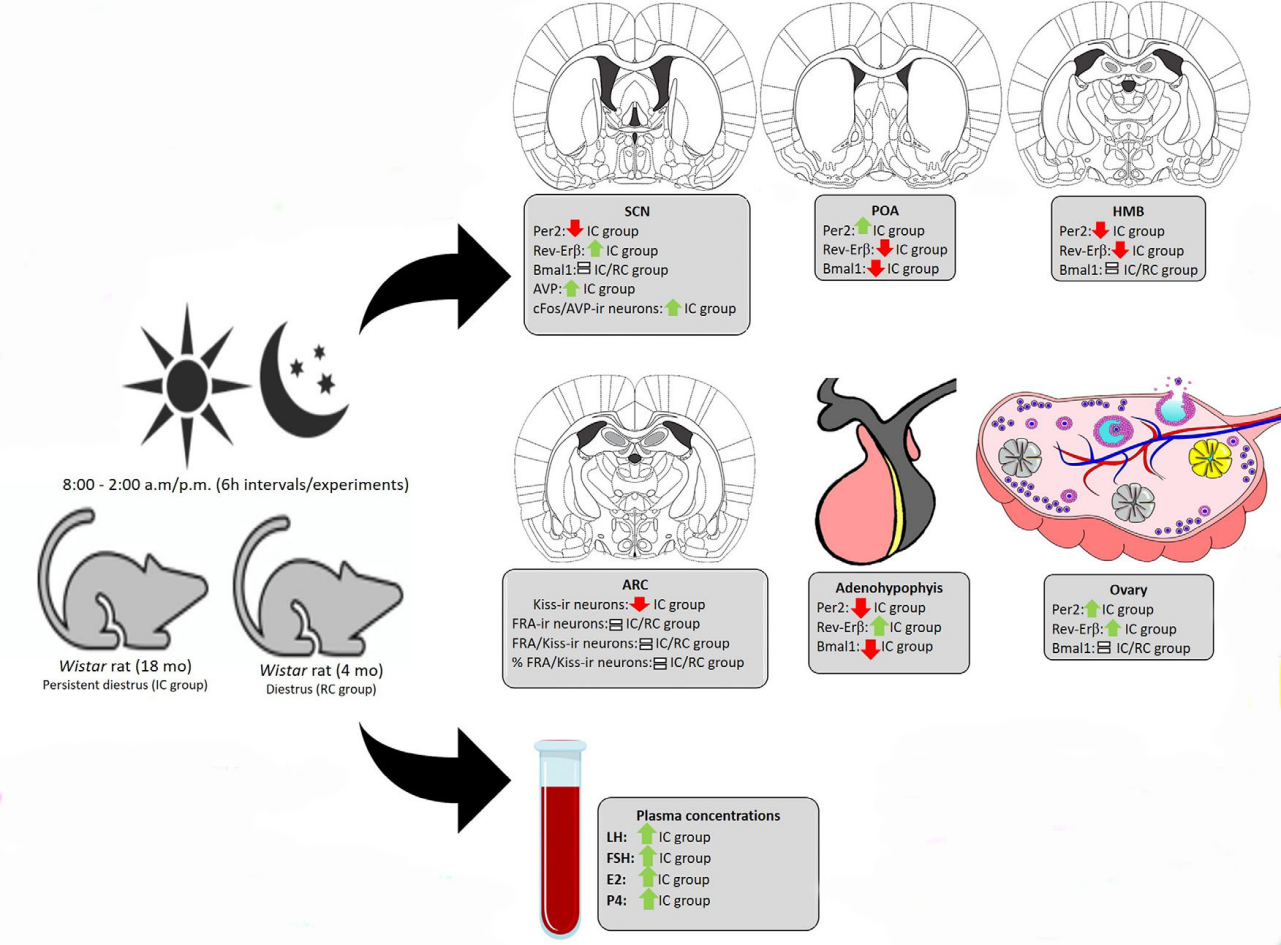
Results summary. Expression profile of clock genes on the HPG axis and activity of SCN AVP neurons and ARC Kiss neurons of rats in periestropause.

Together, these data support our hypothesis that the feedback loops of clock genes on the HPG axis contribute to modulating the spontaneous transition from regular to irregular cycle and to acyclicity in female rodents (**Figure 9**). Additionally, it was evidenced that the desynchronization between the central and peripheral circadian clocks contribute to the irregularity of reproductive events.

## Data Availability Statement

The original contributions presented in the study are publicly available. This data can be found at: http://hdl.handle.net/11449/152720 and https://repositorio.unesp.br/bitstream/handle/11449/152720/nicola_ml_dr_araca_int.pdf?sequence=3&isAllowed=y.

## Ethics Statement

The animal study was reviewed and approved by Ethics Committee of the Universidade Estadual Paulista (Process n. 2014-00269).

## Author Contributions

AN: care of experimental animals, data collection, data analysis, data tabulation, discussion of results, and paper editing. LF: data collection. MM: gene expression data collection and analysis of results. TV-F: standardization of the cFos/AVP immunohistochemistry analysis in SCN nucleus. CL: standardization of the FRA/Kiss immunohistochemistry and data analysis in Arcuate nucleus. AM: FRA/Kiss data analysis in Arcuate nucleus. JA-R: standardization of gene expression and cFos/AVP immunohistochemistry. MP: data analysis, discussion of results, and paper editing. RD: guidance and monitoring of all experimental steps, data analysis, data tabulation, discussion of results, and paper editing. All authors contributed to the article and approved the submitted version.

## Funding

This study was financed in part by the Coordenação de Aperfeiçoamento de Pessoal de Nível Superior—Brasil (CAPES) Finance Code 001 and São Paulo State Research Foundation (FAPESP: 2012/14464-6).

## Conflict of Interest

The authors declare that the research was conducted in the absence of any commercial or financial relationships that could be construed as a potential conflict of interest.

## Publisher’s Note

All claims expressed in this article are solely those of the authors and do not necessarily represent those of their affiliated organizations, or those of the publisher, the editors and the reviewers. Any product that may be evaluated in this article, or claim that may be made by its manufacturer, is not guaranteed or endorsed by the publisher.
